# Dobutamine reverses the vasopressin-associated impairment in cardiac index and systemic oxygen supply in ovine endotoxemia

**DOI:** 10.1186/cc5065

**Published:** 2006-10-10

**Authors:** Christian Ertmer, Andrea Morelli, Hans-Georg Bone, Henning Dirk Stubbe, Ralf Schepers, Hugo Van Aken, Matthias Lange, Katrin Bröking, Martin Lücke, Daniel L Traber, Martin Westphal

**Affiliations:** 1 Department of Anesthesiology and Intensive Care, University Hospital of Muenster, Albert-Schweitzer-Strasse 33, 48149 Muenster, Germany; 2 Department of Anesthesiology and Intensive Care, University of Rome 'La Sapienza', 00185 Rome, Italy; 3 Central Animal Research Facility, University Hospital of Muenster, Muenster, Germany, University Hospital of Muenster, Albert-Schweitzer-Strasse 33, 48149 Muenster, Germany; 4 Investigational Intensive Care Unit, University of Texas Medical Branch, 301 University Boulevard, Galveston TX 77555, USA

## Abstract

**Introduction:**

Arginine vasopressin (AVP) is increasingly used to treat sepsis-related vasodilation and to decrease catecholamine requirements. However, AVP infusion may be associated with a marked decrease in systemic blood flow and oxygen transport. The purpose of the present study was to evaluate whether dobutamine may be titrated to reverse the AVP-related decrease in cardiac index (CI) and systemic oxygen delivery index (DO_2_I) in an established model of ovine endotoxemia.

**Methods:**

Twenty-four adult ewes were chronically instrumented to determine cardiopulmonary hemodynamics and global oxygen transport. All ewes received a continuous endotoxin infusion that contributed to a hypotensive-hyperdynamic circulation and death of five sheep. After 16 hours of endotoxemia, the surviving ewes (*n *= 19; weight 35.6 ± 1.5 kg (mean ± SEM)) were randomized to receive either AVP (0.04 Umin^-1^) and dobutamine (*n *= 8) or the vehicle (normal saline; *n *= 6) and compared with a third group treated with AVP infusion alone (*n *= 5). Dobutamine infusion was started at an initial rate of 2 μg kg^-1^min^-1 ^and was increased to 5 and 10 μg kg^-1 ^min^-1 ^after 30 and 60 minutes, respectively.

**Results:**

AVP infusion increased mean arterial pressure (MAP) and systemic vascular resistance index at the expense of a markedly decreased CI (4.1 ± 0.5 versus 8.2 ± 0.3 l min^-1 ^m^-2^), DO_2_I (577 ± 68 versus 1,150 ± 50 ml min^-1 ^m^-2^) and mixed-venous oxygen saturation (S_v_O_2_; 54.5 ± 1.8% versus 69.4 ± 1.0%; all *p *< 0.001 versus control). Dobutamine dose-dependently reversed the decrease in CI (8.8 ± 0.7 l min^-1 ^m^-2 ^versus 4.4 ± 0.5 l min^-1 ^m^-2^), DO_2_I (1323 ± 102 versus 633 ± 61 ml min^-1 ^m^-2^) and S_v_O_2 _(72.2 ± 1.7% versus 56.5 ± 2.0%, all *p *< 0.001 at dobutamine 10 μg kg^-1 ^min^-1 ^versus AVP group) and further increased MAP.

**Conclusion:**

This study provides evidence that dobutamine is a useful agent for reversing the AVP-associated impairment in systemic blood flow and global oxygen transport.

## Introduction

Septic shock is the most common cause of death in non-coronary intensive care units [1] mainly as a result of catecholamine-refractory arterial hypotension and multiple organ failure. Arginine vasopressin (AVP) is emerging as a promising adjunct in the treatment of catecholamine-refractory septic shock. In this regard, AVP may be administered either as endocrine support targeting to (re)establish adequate AVP plasma levels [2] or as a vasopressor agent seeking to increase mean arterial pressure (MAP) [3]. However, the exact values for 'adequate' AVP plasma levels in endocrine support have not yet been defined.

The hemodynamic state of patients with septic shock treated with aggressive volume challenge is usually characterized by a hyperdynamic circulation, as indicated by increases in cardiac index (CI) and heart rate (HR) and decreases in MAP and systemic vascular resistance index (SVRI). As tissue oxygen requirements are typically increased in patients with septic shock, one of the principal treatment strategies is to maintain high cardiac output and a balanced oxygen supply–demand relationship [4,5]. In contrast, establishing supranormal oxygen delivery has shown inconsistent results and is thus not recommended by the current sepsis guidelines [6,7].

Especially when used in higher doses, AVP may decrease systemic and regional blood flow, thereby impairing tissue oxygen supply [3,8-10]. The latter condition may potentially increase the risk for a so-called 'oxygen supply dependency' and foster the pathogenesis of organ failure or even death. Given that AVP decreases systemic oxygen delivery index (DO_2_I), it seems rational to combine AVP with an inotropic agent that is able to reverse the decrease in CI and DO_2_I.

Whereas some inotropic drugs, such as dopexamine and milrinone, consistently decrease MAP and therefore carry the risk of further decreasing organ perfusion in septic shock [11,12], dobutamine either increases MAP or leaves it unchanged in normovolemic subjects [13].

We hypothesized that dobutamine is a useful agent for decreasing the AVP-associated decreases in systemic blood flow and global oxygen transport in ovine endotoxemia. The present study was conducted to evaluate whether titrated dobutamine is suitable to reverse decreases in CI, DO_2_I and mixed-venous oxygen saturation (S_v_O_2_) resulting from sole AVP infusion in unanesthetized endotoxemic sheep.

## Materials and methods

After study approval by the Local Animal Research Committee, 24 adult ewes were chronically instrumented to determine cardiopulmonary hemodynamics and global oxygen transport with the use of an established protocol [8,11,14-16].

### Animal preparation

Induction of anesthesia was performed by intramuscular injection of S-ketamine (Ketanest 50, 10 mg kg^-1^; Parke-Davis, Berlin, Freiburg, Germany) and xylazine 2% (Xylazin, 0.15 mg kg^-1^; CEVA Tiergesundheit GmbH, Düsseldorf, Germany). Thereafter, anesthesia was maintained with a continuous intravenous infusion of propofol (Disoprivan, 4 to 6 mg kg^-1 ^h^-1^; AstraZeneca, Schwetzigen, Germany). The unconscious, spontaneously breathing ewes were instrumented with an indwelling pulmonary artery catheter, which was inserted by means of the right jugular vein through an introducer sheath (8.5 Fr. Catheter Introducer Set; pvb Medizintechnik GmbH, Kirchseeon, Germany; 7.5 Fr. Edwards Swan Ganz; Edwards Critical Care Division, Irvine, CA, USA) and a left femoral arterial catheter (18-gauge Leader Catheter; Vygon, Aachen, Germany). In addition, a Foley catheter (Porgès S.A., Le Plessis Robinson-Cedex, France) was placed into the urinary bladder to monitor urine output. Intravenous Ceftriaxone (Rocephin 1 g; Hoffmann-La Roche AG, Grenzach-Wyhlen, Germany) was administered as post-surgical infection prophylaxis.

Instrumentation was followed by a 24 hour period of recovery. To prevent postoperative dehydration, all sheep received a continuous intravenous infusion of lactated Ringer's solution (2 ml kg^-1 ^h^-1^).

### Measurement equipment and determined variables

Intravascular catheters were connected to a physiological recorder (Hellige Servomed; Hellige, Freiburg, Germany) by means of pressure transducers (DTX pressure transducer; Ohmeda, Erlangen, Germany). Hemodynamic monitoring included MAP, mean pulmonary arterial pressure (MPAP), central venous pressure, and pulmonary artery occlusion pressure (PAOP). HR was determined by calculating the mean frequency of arterial pressure curve peaks. Core body temperature (*T*) was continuously measured by the thermistor positioned at the tip of the pulmonary artery catheter. The thermodilution technique (9520A cardiac output computer; Edward Lifescience, Irvine, CA, USA) was applied to measure cardiac output by threefold central venous injection of 10 ml of physiological saline solution at a temperature of 2 to 5°C. CI, SVRI, pulmonary vascular resistance index (PVRI), stroke volume index, and left and right ventricular stroke work indices (LVSWI and RVSWI, respectively) were determined with standard equations [11].

Arterial and mixed venous blood samples (0.5 ml each) were collected in heparinized tubes designed for the determination of blood gases (Sarstedt, Nümbrecht, Germany). Partial pressures of O_2 _and CO_2 _(pO_2 _and pCO_2_, respectively) as well as pH were determined with an ABL 725 blood gas analyzer with SAT 100 calibration (Radiometer Copenhagen, Copenhagen, Denmark). In addition, hemoglobin concentration, arterial oxygen saturation (S_a_O_2_), S_v_O_2_, and arterial lactate concentrations were assessed. Standard bicarbonate (HCO_3 _^-^) and base excess (BE) were calculated from pCO_2 _and pH. DO_2_I, oxygen consumption index (VO_2_I) and oxygen extraction rate (O_2_-ER) were determined with standard formulae [11]. All measurements were performed in accordance with the experimental protocol.

### Experimental protocol

Inclusion criteria for the present study were an initial HR of less than 100 beats min^-1^, a core body temperature of 39.8°C or less, a MPAP of less than 25 mmHg and an arterial lactate concentration of one mmol l^-1 ^or less.

During the experimental protocol, all ewes were breathing spontaneously and were studied in a conscious state. Animals were housed in metabolic cages with free access to water and food.

After obtaining baseline cardiopulmonary and oxygen transport data (T1), a hypotensive–hyperdynamic circulation was induced and maintained by a continuous infusion of *Salmonella typhosa *endotoxin (10 ng kg^-1 ^min^-1^; Sigma Chemicals, Deisenhofen, Germany) for the next 18.5 hours. At the same time as endotoxin infusion was started, lactated Ringer's solution was increased from 2 to 4 ml kg^-1 ^h^-1^. Previous studies had demonstrated that this approach keeps PAOP, central venous pressure (CVP) and stroke volume index (SVI) at baseline and guarantees normovolemia of the animals [15]. During the first 14 hours of endotoxemia, five sheep died as a result of right heart failure and were excluded from the study. In the surviving animals (*n *= 19), cardiopulmonary and oxygen transport data were determined after 16 hours of a continuous endotoxin infusion (T2). Thereafter, sheep were randomly allocated to either receive AVP and dobutamine (AVP-Dobu group; *n *= 8) or the vehicle (control group; *n *= 6) and compared with a third group treated with AVP infusion alone (AVP group; *n *= 5). The AVP and AVP-Dobu group received a continuous AVP infusion (Pitressin™ 0.04 U min^-1^; Parke Davis Ltd, Berlin, Freiburg, Germany). After one hour, dobutamine (Dobutamin Liquid Fresenius; Fresenius Kabi, Bad Homburg, Germany) was simultaneously administered at incremental doses in the AVP-Dobu group. Dobutamine infusion was started at a rate of 2 μg kg^-1 ^min^-1 ^and increased to 5 and 10 μg kg^-1 ^min^-1 ^after 30 and 60 minutes, respectively. The control group received only the vehicle (normal saline). Hemodynamic variables and oxygen transport data were analyzed after one hour of AVP infusion (T3), as well as 30 minutes after each dose of dobutamine (T4 to T6). Measurements in the control and AVP group were made at the corresponding time points.

At the end of the experiment the surviving ewes were deeply anesthetized with propofol (4 mg kg^-1^) and killed with a lethal dose of 100 ml potassium chloride solution (7.45%).

### Statistical analysis

Data are expressed as means ± SEM. Sigma Stat 3.10 software (SPSS, Chicago, IL, USA) was used for statistical analysis. After confirming normal distribution of all variables (Kolmogorov–Smirnov test), differences within and between groups were analyzed with a two-way analysis of variance (ANOVA) for repeated measurements. After confirming significant group differences over time, appropriate post hoc comparisons (Student–Newman–Keuls) were performed. For all statistical tests, an error probability of *p *< 0.05 was regarded as statistically significant.

## Results

**Figure 1:**
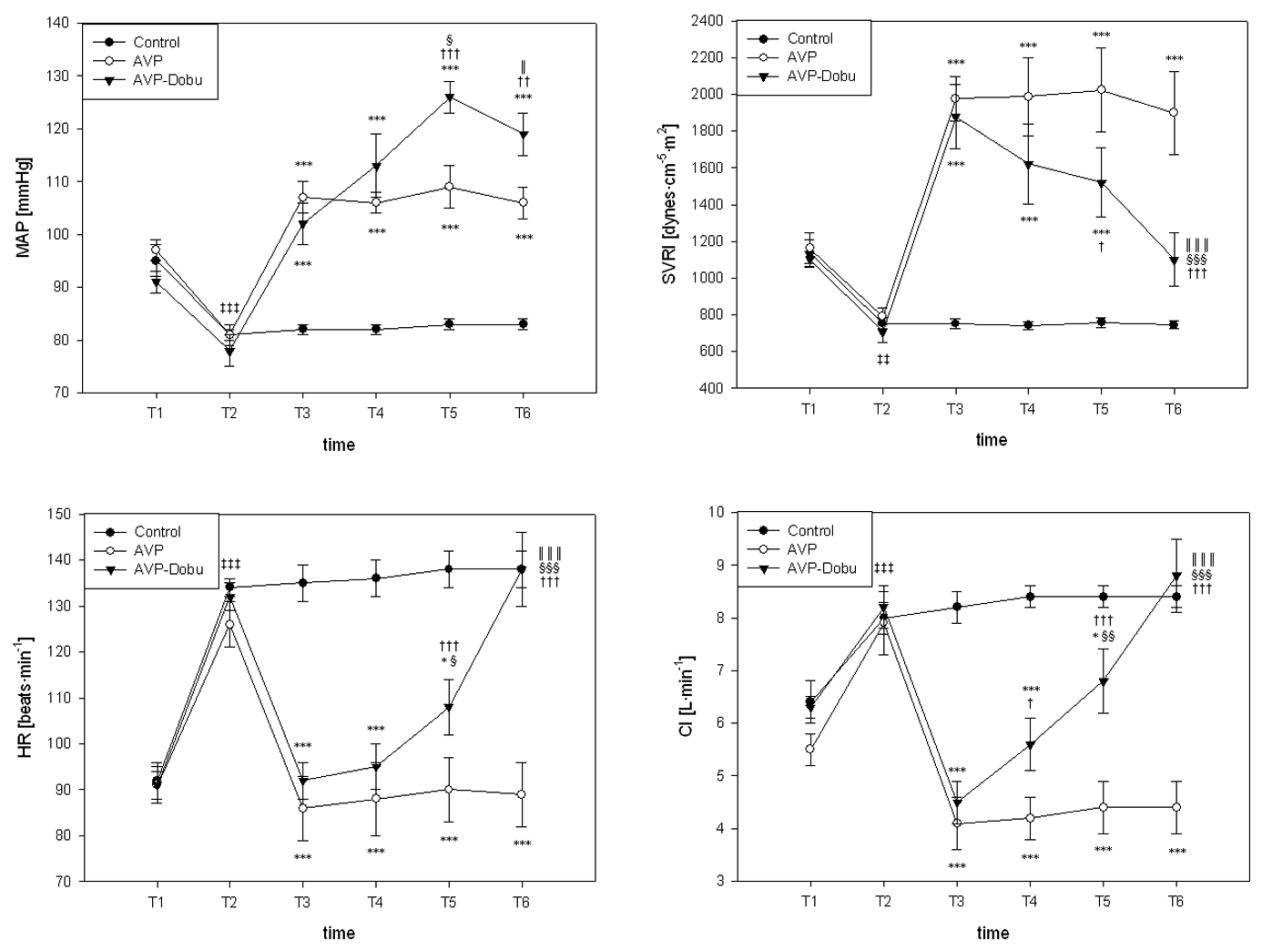
Changes in mean arterial pressure (MAP), systemic vascular resistance index (SVRI), heart rate (HR) and cardiac index (CI). AVP, arginine vasopressin; AVP-Dobu, group treated with AVP and dobutamine; T1, healthy baseline; T2, endotoxemic baseline; T3, AVP or placebo; T4, T5, T6, AVP + dobutamine 2, 5 and 10 μg kg^-1^·min^-1 ^or placebo, respectively. *p < 0.05 versus control, ***p < 0.001 versus control, ^†^*p *< 0.05 versus AVP, ^††^*p *< 0.01 versus AVP, ^†††^*p *< 0.001 versus AVP, ^‡‡‡^*p *< 0.001 versus T1, ^§^*p *< 0.05 versus T4, ^§§^*p *< 0.01 versus T4, ^§§§^*p *< 0.001 versus T4, ^∥^*p *< 0.05 versus T5, ^∥∥∥^*p *< 0.001 versus T5.

**Figure 2:**
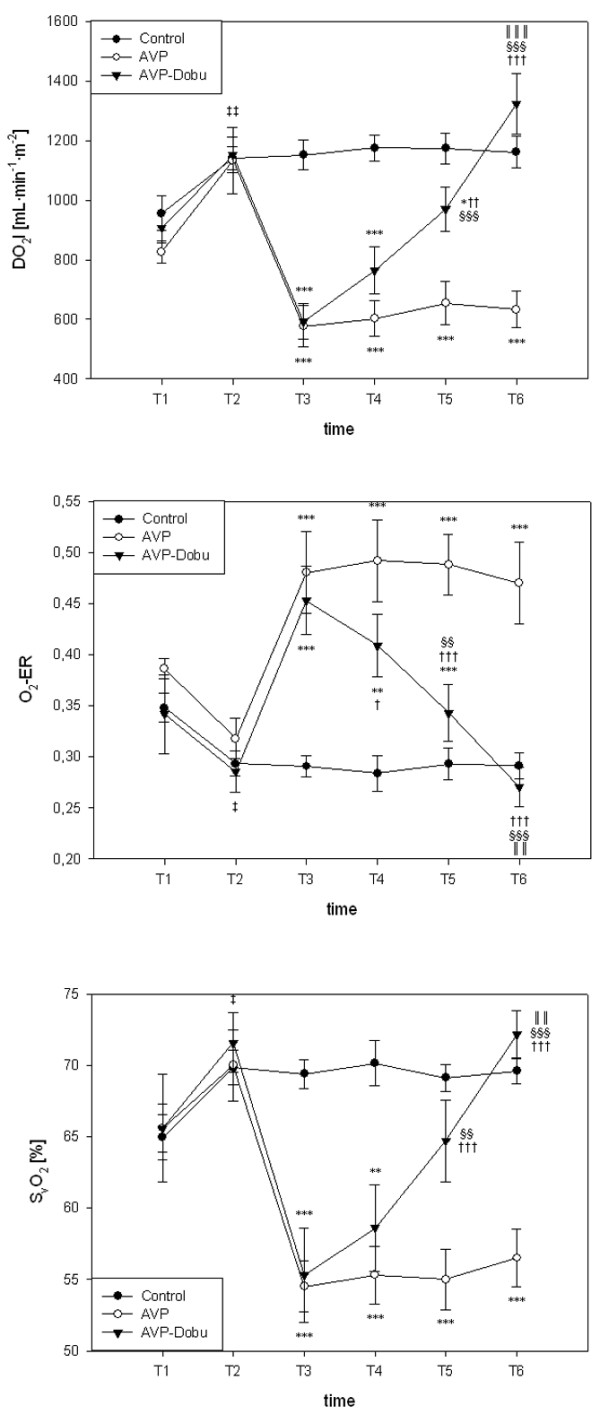
Changes in oxygen delivery index (DO_2_I), oxygen extraction rate (O_2_-ER) and mixed-venous oxygen saturation (S_v_O_2_). AVP, arginine vasopressin; AVP-Dobu, group treated with AVP and dobutamine; T1 = healthy baseline, T2 = endotoxemic baseline, T3 = AVP or placebo, T4, T5, T6 = AVP + dobutamine 2, 5 and 10 μg.kg^-1^·min^-1 ^or placebo, respectively, *p < 0.05 versus control, **p < 0.01 versus control, ***p < 0.001 versus control, ^†^*p *< 0.05 versus AVP, ^††^*p *< 0.01 versus AVP, ^†††^*p *< 0.001 versus AVP, ^‡^*p *< 0.05 versus T1, ^‡‡^*p *< 0.01 versus T1, ^§§^*p *< 0.01 versus T4, ^§§§^*p *< 0.001 versus T4, ^∥∥^*p *< 0.01 versus T5, ^∥∥∥^*p *< 0.001 versus T5.

The entire experiment was performed in 19 sheep with an average weight of 35.6 ± 1.5 kg. Hemodynamic and global oxygen transport variables before endotoxin infusion (T1) are presented in Figures 1 and 2 and Tables 1, 2, 3. There were no statistical differences between groups at randomization.

In all groups, hemoglobin concentration, CVP and PAOP remained constant throughout the entire experiment (Tables 1 and 3).

### Effects of endotoxin infusion

Endotoxin infusion contributed to a hypotensive–hyperdynamic circulation characterized by decreases in MAP (*p *= 0.012 versus healthy state, T1) and SVRI (*p *= 0.02 versus healthy state, T1) as well as increases in HR and CI (each *p *< 0.001 versus healthy state, T1; Figure 1). In comparison with healthy sheep, LVSWI was significantly decreased after 16 hours of endotoxemia (*p *= 0.032; Table 1).

All endotoxemic ewes suffered from pulmonary hypertension, as indicated by increases in MPAP (*p *< 0.001) and PVRI (*p *= 0.042) as compared with the healthy state (T1; Table 1).

In addition, endotoxin infusion contributed to increases in DO_2_I (*p *= 0.003) and S_v_O_2 _(*p *= 0.04) that were accompanied by a decrease in O_2_-ER (*p *= 0.026; all versus healthy state, T1; Figure 2).

In comparison with the healthy state, arterial lactate concentration and core body temperature were elevated (*p *< 0.001) without affecting acid–base balance (Table 2). Urinary output was not significantly altered by endotoxin infusion but tended to increase (Table 3).

There were no statistical differences between groups at T2.

### Effects of AVP infusion in the AVP group

AVP infusion reversed the endotoxin-associated hypotensive–hyperdynamic circulation, as indicated by a decrease in HR and CI and an increase in MAP and SVRI (each *p *< 0.001 versus control; Figure 1).

Infusion of AVP resulted in a further increase in PVRI (*p *= 0.046 versus control) that was associated with a significant decrease in RVSWI (*p *= 0.047 versus control; Table 1).

In addition, AVP infusion led to a marked decrease in DO_2_I, which was accompanied by a sustained increase in O_2_-ER and a decrease in S_v_O_2 _(each *p *< 0.001 versus control; Figure 2). In this study, AVP had no significant impact on VO_2_I but tended to decrease it. Acid–base variables remained constant (Table 2).

Urinary output was markedly increased by AVP infusion (*p *< 0.001 versus control; Table 3).

There was no difference between the AVP and AVP-Dobu group at the time of AVP infusion alone (T3).

### Effects of dobutamine infusion

Dobutamine increased MAP (*p *= 0.009), CI (*p *< 0.001), HR (*p *< 0.001), and LVSWI (*p *= 0.018) and decreased SVRI (*p *< 0.001; all AVP-Dobu versus AVP group at dobutamine 10 μg kg^-1 ^min^-1^; T6) in a dose-dependent manner (Figure 1). In addition, the AVP-associated increase in PVRI was attenuated by dobutamine (*p *= 0.064; AVP-Dobu versus AVP group at dobutamine 10 μg kg^-1 ^min^-1^; T6; Table 1).

Whereas O_2_-ER was markedly decreased after dobutamine infusion, DO_2_I and S_v_O_2 _were significantly increased (each *p *< 0.001; AVP-Dobu versus AVP group at dobutamine 10 μg kg^-1 ^min^-1^; T6; Figure 2). Dobutamine had no effect on VO_2_I and acid–base balance. Similarly, urinary output did not change in comparison with the AVP group (Table 3).

Dobutamine-related effects were dose-dependent and most pronounced at the highest dosage (namely 10 μg kg^-1 ^min^-1^; T6).

## Discussion

In the present study the effects of a titrated dobutamine infusion on cardiopulmonary hemodynamics and global oxygen transport were evaluated in endotoxemic sheep treated with a fixed AVP infusion (0.04 U min^-1^). The major finding is that dobutamine reversed the AVP-associated impairment in CI, DO_2_I and S_v_O_2 _in a dose-dependent manner.

To our knowledge, this is the first study elucidating the interactions between AVP and titrated dobutamine in awake animals suffering from chronic endotoxemia. Martikainen and colleagues have already reported that dobutamine compensates for deleterious hemodynamic and metabolic effects of AVP in the splanchnic region in endotoxic shock in anesthetized, continuously ventilated domestic pigs [17]. However, it is noteworthy that the latter authors used almost twice the dosage of AVP (0.002 U kg^-1 ^min^-1^) than we did in the present study (about 0.001 U kg^-1 ^min^-1^). Whereas only low-dose dobutamine (2.8 μg kg^-1 ^min^-1^) was infused in the experiment by Martikainen and colleagues [17], the present study investigated the effects of different doses.

Notably, we used a large animal model that closely reflects hemodynamic changes seen in septic patients with a hyperdynamic circulation [18,19]. In harmony with previous studies using the same or similar sepsis models [8,11,14-16,20], endotoxin infusion was linked to a decrease in vascular resistance and MAP as well as an increase in CI, and was accompanied by elevations in core body temperature and arterial lactate concentrations.

In the present study, AVP was used in a moderate dosage (0.04 U min^-1^), seeking to reverse endotoxin-induced vasodilation and arterial hypotension. In accordance with previous studies, AVP infusion was linked to substantial vasoconstriction, as reflected by a significant increase in SVRI [8,15]. The mechanisms of this finding include, but may not be restricted to, activation of vascular V_1 _receptors [21], inhibition of NO-mediated cyclic GMP production [22] and inhibition of vascular ATP-controlled potassium channels (K_ATP _channels) [23].

The AVP-induced decrease in HR may be explained by baroreceptor activation and is in line with previous experimental and clinical studies [8,21,24]. The subsequent decrease in CI was associated with a proportional decrease in DO_2_I. To maintain VO_2_I above critical threshold values, O_2_-ER had to be increased. Nevertheless, VO_2_I tended to decrease in the AVP-treated groups.

In this context, it should be kept in mind that a marked decrease in DO_2_I carries the risk for impaired regional oxygen supply, especially of the gastrointestinal tract. As a result of increased mucosal oxygen consumption in patients with sepsis [25], a decrease in oxygen delivery may impair the gut mucosal barrier, thereby leading to bacterial translocation and fostering the inflammatory septic cascade [26]. Strategies to prevent an AVP-associated impairment in DO_2_I therefore seem to be of significant clinical relevance.

Dobutamine is a partial agonist on β_1_- and β_2_-adrenoceptors with little effect on α-adrenoceptors, and increases HR, CI and DO_2_I within a therapeutic range of 1 to 20 μg kg^-1 ^min^-1^. In normovolemic subjects the increase in CI is associated with no change or an increase in systemic blood pressure. In contrast, in the presence of hypovolemia, dobutamine may increase myocardial oxygen demand and decrease MAP [13]. In the present study, dobutamine caused dose-dependent increases in MAP, CI, HR, DO_2_I and S_v_O_2_, thereby improving both systemic hemodynamics and global oxygen transport. Our group previously reported that dopexamine, a synthetic catecholamine with intrinsic activity on dopaminergic DA_1 _and DA_2 _receptors as well as on β_1_- and β_2_-adrenoceptors, increases HR, CI and LVSWI in AVP-treated endotoxemic sheep [11]. However, probably because of the vasodilating action through vascular DA_1_- and β_2_-receptors, dopexamine decreased MAP, thereby limiting its therapeutic use.

Dobutamine is currently the inotropic agent of choice to increase CI in patients with septic shock with an inappropriately low cardiac output [7,27]. The present study provides evidence that dobutamine may also be suitable for reversing the AVP-related impairment in CI, DO_2_I and S_v_O_2_. In addition, dobutamine decreased SVRI to values noticed before injury and improved LVSWI, a marker of myocardial contractility. However, it must be considered that dobutamine increased HR to values noticed before AVP infusion and may therefore potentially bear the risk of adverse cardiac events, such as tachyarrhythmias and myocardial ischemia [28]. Although the AVP-associated decreases in HR, CI and DO_2_I seem critical, no clinical study has yet shown an impaired outcome due to these AVP-related side effects. Conversely, no study has ever shown benefit from elevating HR, CI and DO_2_I in AVP-treated patients. Nevertheless, it is noteworthy that an early goal-directed therapy seeking to establish a S_v_O_2 _of more than 70% has proven to decrease mortality in patients with septic shock [29].

The present study has some limitations that we acknowledge. First, we used an animal model to mimic hemodynamics in human sepsis. In harmony with previous studies of our group, endotoxemic sheep suffered from moderate arterial hypotension (MAP 82 ± 2 mmHg) [8,11,14-16]. In this context, however, it is important that sheep are physiologically characterized by higher blood pressures than humans. A decrease in MAP from 100 to about 80 mmHg is a typical feature of ovine endotoxemia, which represents one of the most frequently used animal models in investigating vasoactive substances for the treatment of sepsis. Consequently, studies using the same or similar sheep models resulted in comparable hemodynamic variables [8,11,14-16,20]. Dose-response studies in sheep with higher doses of endotoxin did not result in a MAP of less than 55 mmHg unless the animals died (Ertmer C, 2006, unpublished observations). In addition, the marked decrease in SVRI by endotoxin infusion reflects pronounced vasodilation, similar to what can be observed in human septic shock [30].

Because we did not investigate regional blood flow and oxygen supply of distinct organs, it can only be speculated that the AVP-associated decrease in CI was associated with impaired tissue oxygen supply. However, previous clinical and experimental studies clearly suggest that an AVP-induced decrease in CI may contribute to hypoperfusion of splanchnic organs [9,10,31].

Finally, we emphasize that it was not the aim of the present study to encourage the use of AVP as a single first-line vasopressor, but to determine the effects of dobutamine infusion on the AVP-associated decrease in systemic blood flow and global oxygen transport.

## Conclusion

Despite its limitations, this study provides evidence that dobutamine is a useful agent for reversing the AVP-associated depressions in CI and global oxygen supply. Whether a pharmacological increase in CI and S_v_O_2 _improves the overall outcome in human septic shock treated with vasopressin analogues should be addressed in randomized controlled clinical trials.

## Key messages

• AVP impairs the CI and the systemic oxygen supply when used in a moderate dose (0.04 U min^-1^) in ovine endotoxemia.

• In fluid-challenged endotoxemic sheep, dobutamine reverses the AVP-associated impairment in CI, DO_2_I and S_v_O_2_, and further increases MAP.

• The dobutamine-associated effects are dose-dependent and strongest at an infusion rate of 10 μg kg^-1 ^min^-1^.

## Abbreviations

AVP = arginine vasopressin; CI = cardiac index; DO_2_I = oxygen delivery index; HR = heart rate; LVSWI = left ventricular stroke work index; MAP = mean arterial pressure; MPAP = mean pulmonary arterial pressure; O_2_-ER = oxygen extraction rate; PAOP = pulmonary arterial occlusion pressure; PVRI = pulmonary vascular resistance index; RVSWI = right ventricular stroke work index; S_v_O_2 _= mixed-venous oxygen saturation; SVRI = systemic vascular resistance index; VO_2_I = oxygen consumption index.

## Competing interests

The authors declare that they have no competing interests.

## Authors' contributions

CE, RS, HGB, HDS, HVA, ML, KB, DLT and MW contributed to the study design and the acquisition of the data. CE, RC, AM and MW contributed to analyses and interpretation of the data. CE and MW did the main writing of the manuscript. CE, AM and MW were involved in writing and revising the manuscript. ML contributed to the revision of the manuscript. All authors have read, supplemented and given final approval to the manuscript.

## Figures and Tables

**Table 1:** Changes in hemodynamic variables in endotoxemic sheep.

Variable	Group	T1	T2	T3	T4	T5	T6
MPAP (mmHg)	Control	18 ± 1	25 ± 1^‡‡‡^	26 ± 1	26 ± 1	26 ± 1	25 ± 1
	AVP	19 ± 1	27 ± 1^‡‡‡^	27 ± 2	27 ± 2	26 ± 2	27 ± 3
	AVP-Dobu	20 ± 1	25 ± 1^‡‡‡^	25 ± 1	25 ± 1	25 ± 1	24 ± 1
PVRI (dyne.s.cm^-5^·m^2^)	Control	99 ± 8	138 ± 18^‡^	142 ± 14	145 ± 13	146 ± 11	140 ± 10
	AVP	98 ± 12	124 ± 18	184 ± 34*	173 ± 37	179 ± 35	176 ± 35
	AVP-Dobu	107 ± 9	125 ± 12	191 ± 30*	163 ± 16	132 ± 16	133 ± 16
CVP (mmHg)	Control	5 ± 0	7 ± 1	7 ± 0	6 ± 1	6 ± 1	6 ± 1
	AVP	5 ± 1	8 ± 0^‡^	8 ± 1	8 ± 1	7 ± 1	8 ± 1
	AVP-Dobu	6 ± 1	7 ± 1	9 ± 1	8 ± 1	6 ± 1	6 ± 1
PAOP (mmHg)	Control	11 ± 1	12 ± 1	12 ± 1	12 ± 1	12 ± 1	12 ± 1
	AVP	9 ± 0	13 ± 1^‡^	15 ± 1	15 ± 1	15 ± 1	14 ± 1
	AVP-Dobu	11 ± 1	12 ± 2	15 ± 2	15 ± 1	14 ± 1	12 ± 1
SVI (ml beat^-1 ^m^-2^)	Control	69 ± 3	60 ± 3	61 ± 3	62 ± 3	62 ± 3	62 ± 2
	AVP	61 ± 3	62 ± 4	48 ± 5	48 ± 6	47 ± 3	47 ± 3
	AVP-Dobu	70 ± 3	63 ± 4	49 ± 5	59 ± 6	63 ± 5^†^	64 ± 5^††^
LVSWI (g m^-1 ^m^2^)	Control	79 ± 5	57 ± 3^‡^	59 ± 3	61 ± 3	64 ± 3	60 ± 2
	AVP	75 ± 3	57 ± 4^‡^	62 ± 11	62 ± 11	68 ± 12	70 ± 12
	AVP-Dobu	76 ± 4	56 ± 5^‡‡^	59 ± 6	80 ± 9	95 ± 7**††§	94 ± 8**†§
RVSWI (g m^-1 ^m^2^)	Control	12 ± 1	15 ± 1	16 ± 1	17 ± 1	17 ± 1	16 ± 1
	AVP	12 ± 1	16 ± 1	12 ± 2*	11 ± 1*	13 ± 1	13 ± 1
	AVP-Dobu	13 ± 0	15 ± 2	11 ± 2**	14 ± 2	16 ± 2	17 ± 2

AVP, arginine vasopressin; AVP-Dobu, group treated with AVP and dobutamine; MPAP, mean pulmonary arterial pressure; PVRI, pulmonary vascular resistance index; CVP, central venous pressure; PAOP, pulmonary artery occlusion pressure; SVI, stroke volume index; LVSWI, left ventricular stroke work index; RVSWI, right ventricular stroke work index; T1, healthy baseline; T2, endotoxemic baseline; T3, AVP or placebo; T4, T5, T6, AVP + dobutamine 2, 5 and 10 μg kg^-1 ^min^-1 ^or placebo, respectively. *p < 0.05 versus control, **p < 0.01 versus control, ^†^*p *< 0.05 versus AVP, ^††^*p *< 0.01 versus AVP, ^‡^*p *< 0.05 versus T1, ^‡‡^*p *< 0.01 versus T1, ^‡‡‡^*p *< 0.001 versus T1, ^§^*p *< 0.05 versus T4.

**Table 2:** Changes in variables of acid-base balance and arterial lactate in endotoxemic sheep.

Variable	Group	T1	T2	T3	T4	T5	T6
Arterial pH	Control	7.46 ± 0.00	7.47 ± 0.00	7.46 ± 0.00	7.47 ± 0.00	7.47 ± 0.00	7.47 ± 0.00
	AVP	7.45 ± 0.02	7.47 ± 0.02	7.45 ± 0.03	7.46 ± 0.02	7.48 ± 0.02	7.47 ± 0.02
	AVP-Dobu	7.45 ± 0.02	7.47 ± 0.01	7.45 ± 0.01	7.45 ± 0.01	7.45 ± 0.01	7.44 ± 0.01
Arterial BE (mmol l^-1^)	Control	4.4 ± 0.5	4.5 ± 0.9	4.9 ± 1	4.6 ± 1	4.1 ± 1	4.2 ± 1
	AVP	2.4 ± 1.5	4.0 ± 1.0	3.1 ± 1.4	3.8 ± 1.5	3.1 ± 1.3	3.2 ± 1.3
	AVP-Dobu	3.2 ± 0.9	3.2 ± 1	2.3 ± 1	2.1 ± 1.2	1.7 ± 1.2	1.5 ± 1.3
Arterial lactate (mmol l^-1^)	Control	0.7 ± 0.1	1.6 ± 0.2^‡‡‡^	1.6 ± 0.1	1.8 ± 0.2	1.9 ± 0.2	2.1 ± 0.2
	AVP	0.6 ± 0.1	1.4 ± 0.3^‡‡^	1.4 ± 0.3	1.6 ± 0.4	1.6 ± 0.4	1.8 ± 0.4
	AVP-Dobu	0.8 ± 0.1	1.5 ± 0.2^‡‡‡^	1.7 ± 0.3	1.4 ± 0.2	1.6 ± 0.1	2.0 ± 0.2
VO_2_I (ml min^-1 ^m^-2^)	Control	332 ± 20	336 ± 20	335 ± 20	336 ± 19	346 ± 17	339 ± 17
	AVP	317 ± 12	357 ± 37	287 ± 21	287 ± 21	303 ± 25	307 ± 13
	AVP-Dobu	316 ± 16	333 ± 17	265 ± 13	306 ± 17	328 ± 16	361 ± 15
Temperature (°C)	Control	39.5 ± 0.1	41.0 ± 0.2^‡‡‡^	41.0 ± 0.1	41.0 ± 0.2	41.0 ± 0.2	41.0 ± 0.2
	AVP	39.7 ± 0.1	40.7 ± 0.2^‡‡‡^	40.7 ± 0.2	40.7 ± 0.2	40.7 ± 0.1	40.7 ± 0.1
	AVP-Dobu	39.5 ± 0.1	41.0 ± 0.2^‡‡‡^	40.9 ± 0.2	40.9 ± 0.2	40.8 ± 0.1	40.9 ± 0.2

AVP, arginine vasopressin; AVP-Dobu, group treated with AVP and dobutamine; BE, base excess; T1, healthy baseline; T2, endotoxemic baseline; T3, AVP or placebo; T4, T5, T6, AVP + dobutamine 2, 5 and 10 μg kg^-1 ^min^-1 ^or placebo, respectively. ^‡‡^*p *< 0.01 versus T1, ^‡‡‡^*p *< 0.001 versus T1.

**Table 3:** Changes in urinary output and hemoglobin concentration in endotoxemic sheep.

Variable	Group	T1	T2	T3	T4	T5	T6
Urinary output (ml h^-1^)	Control	63 ± 5	94 ± 12	85 ± 13	n/a	n/a	97 ± 17
	AVP	66 ± 12	108 ± 14	246 ± 41***	n/a	n/a	192 ± 22**
	AVP-Dobu	69 ± 8	105 ± 14	264 ± 33***	n/a	n/a	252 ± 24***
Hemoglobin (g dl^-1^)	Control	10.9 ± 0.3	10.5 ± 0.4	10.4 ± 0.3	10.4 ± 0.4	10.3 ± 0.3	10.2 ± 0.4
	AVP	10.5 ± 0.4	10.3 ± 0.3	10.1 ± 0.3	10.2 ± 0.3	10.4 ± 0.4	10.3 ± 0.4
	AVP-Dobu	10.5 ± 0.4	10.2 ± 0.3	9.9 ± 0.3	10.1 ± 0.5	10.7 ± 0.5	10.1 ± 0.5

AVP, arginine vasopressin; AVP-Dobu, group treated with AVP and dobutamine; T1, healthy baseline; T2, endotoxemic baseline; T3, AVP or placebo; T4, T5, T6, AVP + dobutamine 2, 5 and 10 μg kg^-1 ^min^-1 ^or placebo, respectively. ***p < 0.001 versus control.
